# Zinner syndrome: an unusual cause of bladder outflow obstruction

**DOI:** 10.1259/bjrcr.20160094

**Published:** 2017-02-15

**Authors:** Sonali Shah, Ramesh Patel, Rakesh Sinha, Maya Harris

**Affiliations:** ^1^Department of Gastroenterology, South Warwick Hospital, Warwick, UK; ^2^Department of Radiology, South Warwick Hospital, Warwick, UK; ^3^Department of Urology, South Warwick Hospital, Warwick, UK

## Abstract

Zinner syndrome is a rare condition comprising a triad of unilateral renal agenesis, ipsilateral seminal vesicle obstruction and ipsilateral ejaculatory duct obstruction. The mutual embryological origins of the seminal vesicle and the ureteral bud result in both anomalous genital and urinary tracts. We present the case of a 39-year-old patient where the initial presentation of this condition was bladder outflow obstruction. In this paper, we discuss the embryological origin of this condition, the range of imaging tools used to diagnose Zinner syndrome and the inherent benefits and shortcomings of each modality.

## Case presentation

A 39-year-old patient presented with a longstanding history of bladder and voiding problems. The symptoms peaked a year ago following a long lorry drive on the motorway when he developed suprapubic pain followed by terminal dribbling and urinary retention necessitating admission. He had a previous childhood history of recurrent urinary tract infections, nocturia and suprapubic pain when the bladder was full. He had one previous episode of subacute retention 8 years ago requiring catheterization and drained 2 l of urine. The cause of retention was initially thought to be secondary to a large 5 cm bladder diverticulum causing bilateral hydronephrosis seen on CT scan examination. On the same examination, it was noticed that the right kidney cortex was atrophic and the kidney was filled with urine with increased density.

The cause of the recent symptoms was attributed to the previously recorded diagnosis of bladder dysfunction with bilateral hydronephrosis and secondary urinary tract infections. The patient was commenced on alfuzosin and taught intermittent self- catheterization. A flexible cystoscopy was also carried out to determine if there was any prostatic occlusion, which corroborated the tight bladder neck diverticulum as previously described.

## Imaging findings

**Figure 1.**
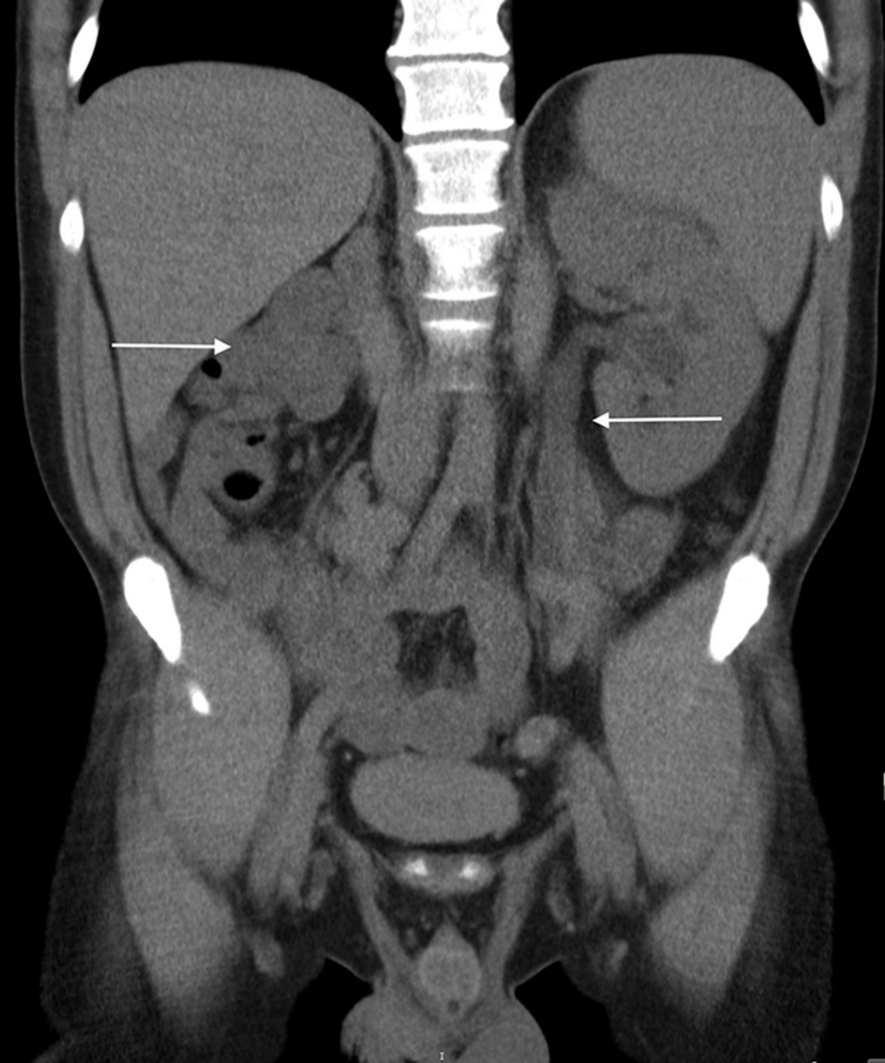
CT coronal view showing hypoplastic right kidney and dilated left ureter and mild hydronephrosis.

**Figure 2.**
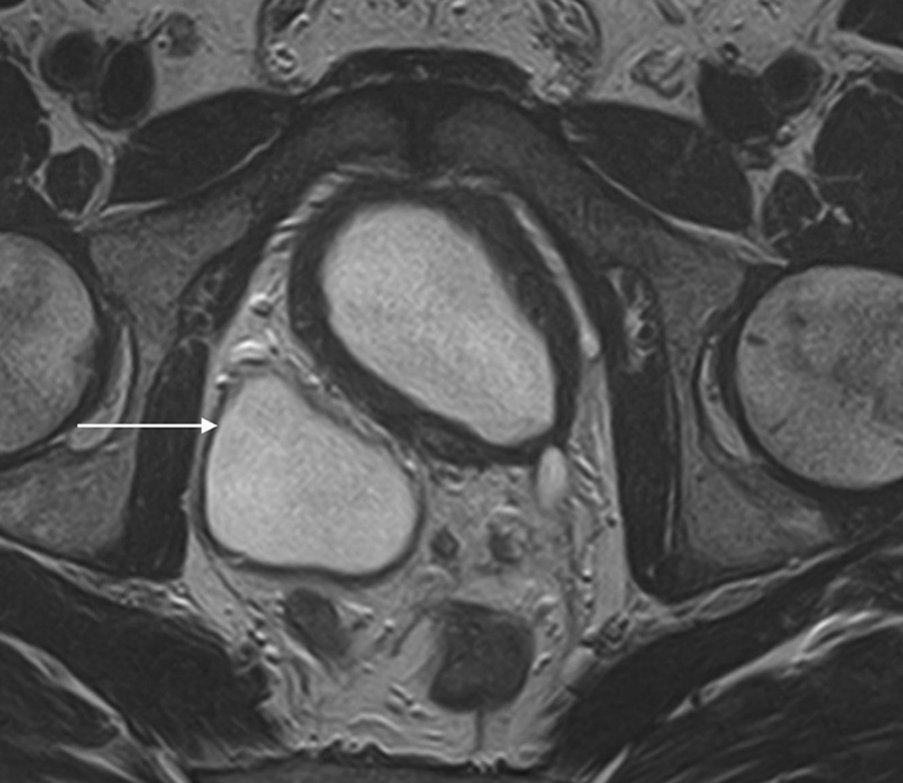
Axial *T*_2_ weighted MRI image showing thickened bladder and dilated, cystic right ureteric bud.

**Figure 3.**
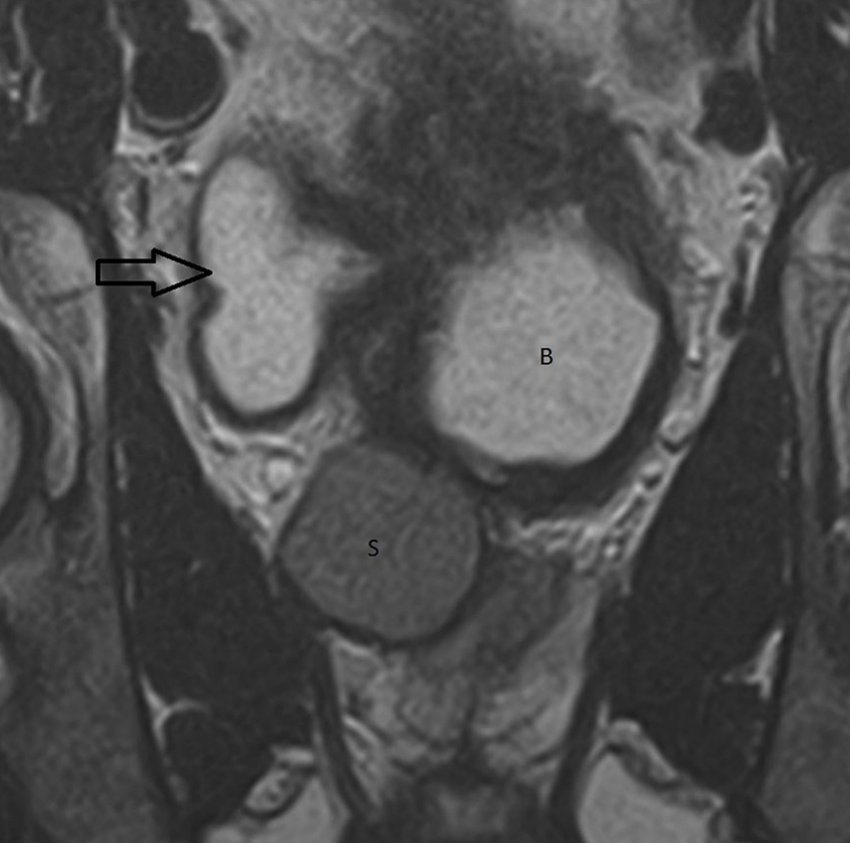
Coronal *T*_2_ weighted MRI image showing thickened bladder (B), cystic right ureteric bud opening in the bladder (arrow) and dilated seminal vesicle (S).

**Figure 4.**
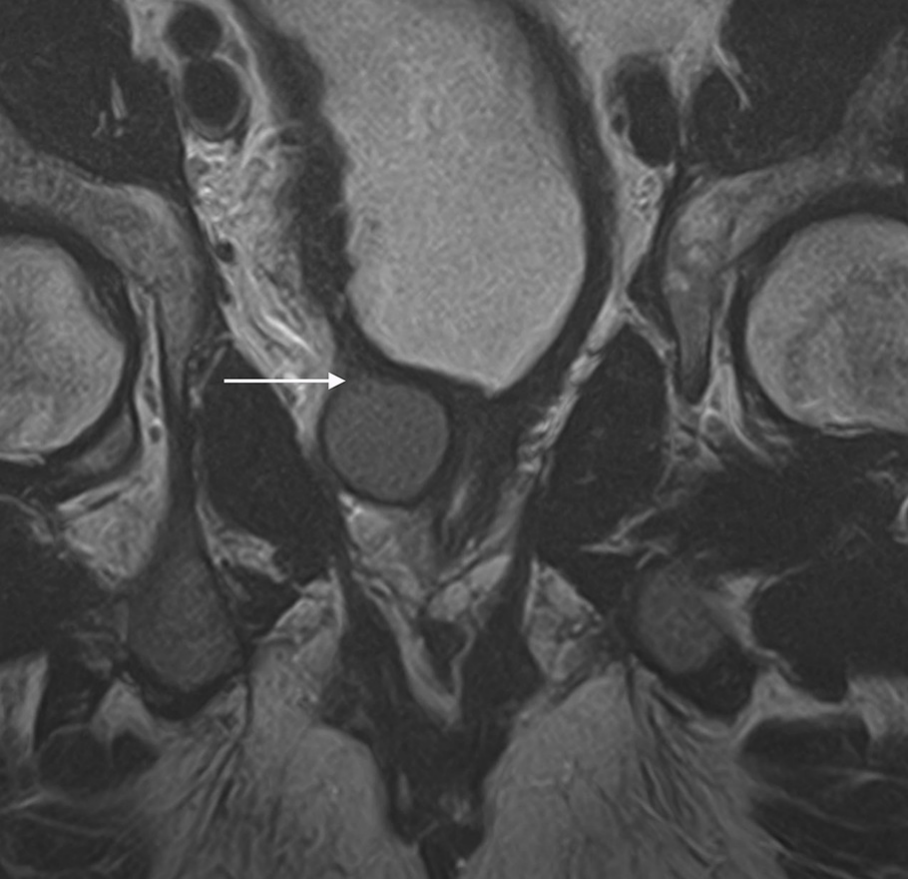
Coronal *T*_2_ weighted MRI image showing seminal vesicle cyst causing occlusion of bladder neck.

**Figure 5.**
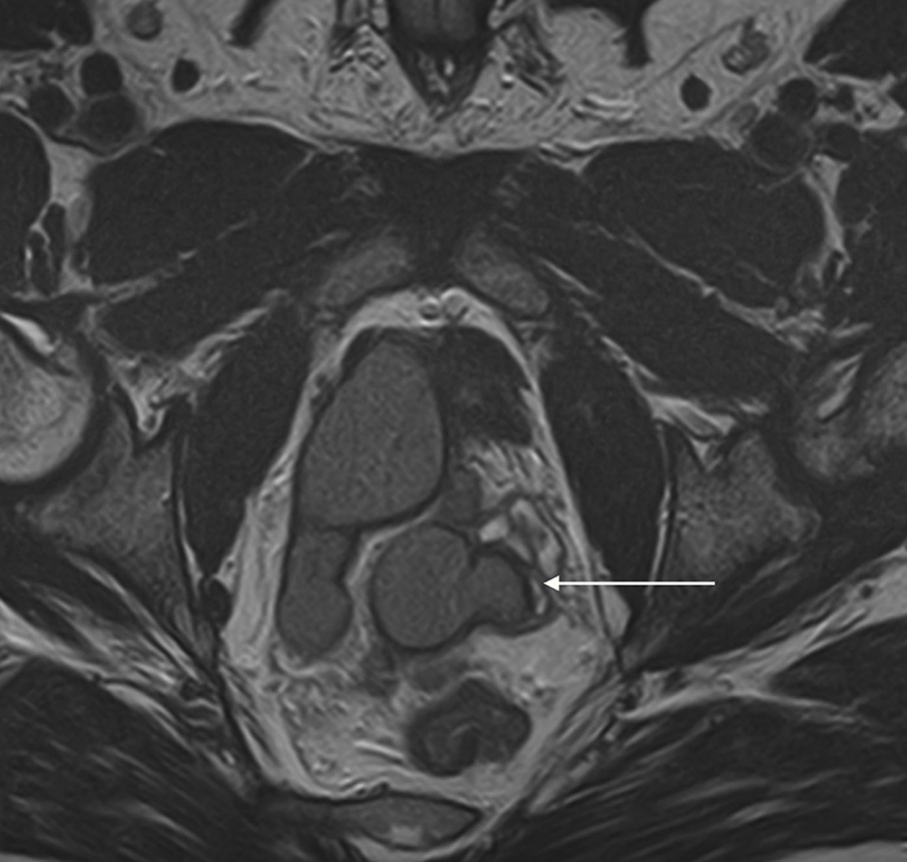
Axial *T*_2_ weighted MRI image showing dilated, tortuous seminal vesicle.

**Figure 6.**
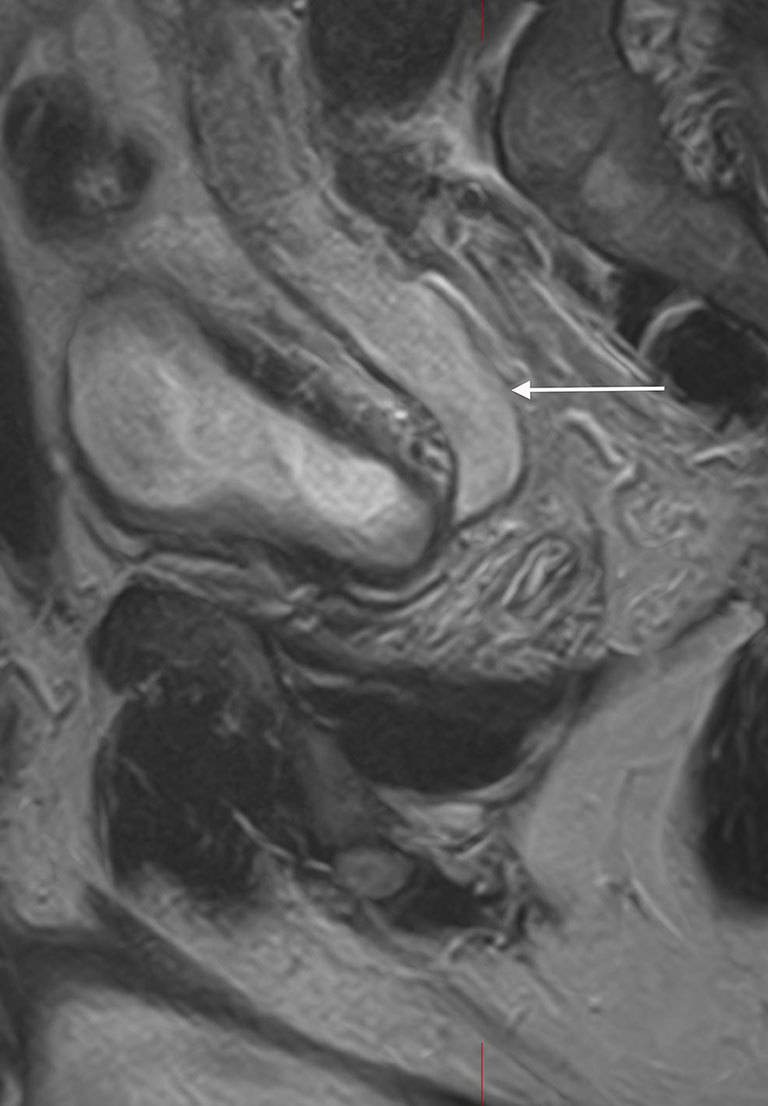
Sagittal *T*_2_ weighted MRI image showing dilated left ureter.

An MRI of the spine showed no pathology related to the spinal cord and no impingement involving the spinal cord or the exiting nerve roots. A CT examination of the urinary tract demonstrated a small atrophic renal remnant in the right renal fossa (Figure 1). In addition, there was ectopic insertion of the right ureter behind the bladder neck associated with cystic ureteric bud (Figures 2,3). An MRI examination of the pelvis was carried out for further evaluation, which demonstrated anomalous dilatation and dysplasia of the right seminal vesicle and ejaculatory duct (Figures 4–6). The triad of renal dysplasia as opposed to agenesis, ipsilateral seminal vesicle cyst and ejaculatory duct obstruction confirmed the diagnosis of a variant of Zinner syndrome.

## Treatment/outcome

The patient underwent endoscopic incision of right seminal vesicle to relieve the obstruction, which drained a significant amount of pus. This alleviated the outflow obstruction and as a result the patient can now void independently and no longer requires self-catheterization. This has remarkably improved his quality of life.

## Discussion

Zinner syndrome is a Wolffian duct abnormality comprising a triad of unilateral renal agenesis, ipsilateral seminal vesicle cyst and ejaculatory duct obstruction. This condition was first described by Zinner in 1914.^1^ This is a rare condition with less than 200 cases reported in the literature. The association between upper urinary tract abnormalities and seminal vesicle malformation are based on the shared origin of the ureteral buds and seminal vesicles from the mesonephric (Wolffian) duct.^2^ This abnormality arises following an insult during the first trimester of embryogenesis.

The ureteral bud originates from the dorsal aspect of the distal mesonephric duct and extends in a dorsocranial manner to meet and induce transformation of the metanephric blastema to form the adult kidney. The mesonephric duct differentiates into the epididymis, paradidymis, vas deferens, ejaculatory duct, seminal vesicle and hemitrigone under the influence of testosterone and anti-mullerian hormone.^3,4^ Therefore, failure of the ureteral bud to meet the metanephric blastema results in renal agenesis of the affected side, while development of the seminal vesicles is preserved. Alternatively, in the context of maldevelopment of the mesonephric duct altogether, there will be abnormal ureteral budding leading to ipsilateral renal agenesis or dysplasia and atresia of the ejaculatory duct with consequent obstruction and cystic dilatation of the seminal vesicles.^5^

Most patients remain asymptomatic till the second to fourth decade of life relating to the period of highest sexual and reproductive activity.^6^ The clinical presentation relates to the size of the seminal vesicles. Cysts less than 5 cm diameter are often diagnosed incidentally on abdominal or digital rectal examination. Symptoms arise following progressive dilation of the seminal vesicles due to accumulation of secretions following insufficient drainage secondary to ejaculatory duct atresia.^2^ The seminal vesicles are located directly posterior to the bladder and therefore enlarged cysts can cause bladder irritation leading to symptoms of dysuria, recurrent urinary tract infections, infertility, painful ejaculation, epididymitis and prostatitis.^7–9^ Large cysts can also cause bladder outlet or colonic obstruction.

A spectrum of imaging techniques may be used in the diagnostic work-up of this condition. These include intravenous urography, transrectal ultrasonography, CT scanning, cystoscopy and MR imaging. Intravenous urography can be used to assess abnormalities or absence of collecting duct system and therefore demonstrate renal agenesis but may overlook lesions extrinsic to the renal tract.^10^ Transrectal sonography can be used to determine the size, location and nature of a cystic mass associated with the seminal vesicle or prostate. It may demonstrate anechoic contents or contain debris suggesting previous haemorrhage or infection but is limited by its small field of view.

CT scanning can accurately depict pelvic anatomy and atypical renal structures. However, the communication between seminal vesicle cysts and adjacent pelvic structures or the urinary tract can be difficult to visualize owing to its relatively poorer contrast resolution compared to MR imaging. MRI is the diagnostic tool of choice owing to its greater tissue contrast resolution allowing detailed pelvic anatomy and differentiation of cystic masses and hence providing definitive diagnosis. Seminal vesicles appear as low attenuation on *T*_1_ weighted images and conversely high attenuation on *T*_2_ weighted images.^11^ In cases where surgical management is appropriate MR imaging can be helpful in surgical planning for seminal vesicle excision.

In this case, MR imaging demonstrated the right distal ureter communicating with a cyst, which then emptied via the vesicourteric junction into the bladder. Abnormalities of the seminal vesicle and the ejaculatory ducts were also demonstrated on the MR images revealing the diagnosis to be a variant of Zinner syndrome.

## Learning points

1. Zinner syndrome is a triad of unilateral renal agenesis, ipsilateral seminal vesicle obstruction and ipsilateral ejaculatory duct obstruction.
2. The mesonephric duct gives rise to the adult kidney as well as differentiates into several structures of the genital tract.
3. Presentation of the syndrome predominantly relates to the size of the seminal vesicle.
4. MR imaging is the modality of choice in order to yield a definitive diagnosis.

## Consent

Written informed consent for the case to be published (including images, case history and data) was obtained from the patient(s) for publication of this case report, including accompanying images.
